# Multiple antigen-engineered DC vaccines with or without IFNα to promote antitumor immunity in melanoma

**DOI:** 10.1186/s40425-019-0552-x

**Published:** 2019-04-24

**Authors:** Lisa H. Butterfield, Lazar Vujanovic, Patricia M. Santos, Deena M. Maurer, Andrea Gambotto, Joel Lohr, Chunlei Li, Jacob Waldman, Uma Chandran, Yan Lin, Huang Lin, Hussein A. Tawbi, Ahmad A. Tarhini, John M. Kirkwood

**Affiliations:** 1 Department of Medicine, University of Pittsburgh, UPMC Hillman Cancer Center, 5117 Centre Avenue, Suite 1.27, Pittsburgh, PA 15213 USA; 2 Department of Surgery, University of Pittsburgh, UPMC Hillman Cancer Center, 5117 Centre Avenue, Suite 1.27, Pittsburgh, PA 15213 USA; 3 Department of Immunology, University of Pittsburgh, UPMC Hillman Cancer Center, 5117 Centre Avenue, Suite 1.27, Pittsburgh, PA 15213 USA; 4 UPMC Hillman Cancer Center, University of Pittsburgh, UPMC Hillman Cancer Center, 5117 Centre Avenue, Suite 1.27, Pittsburgh, PA 15213 USA; 5 Department of Biomedical Informatics, University of Pittsburgh, Pittsburgh, PA USA; 6 Department of Biostatistics, University of Pittsburgh, Pittsburgh, PA USA; 7 Present address: Tsinghua University School of Medicine, Beijing, China; 8 Present address: Department of Melanoma Medical Oncology, University of Texas MD Anderson Cancer Center, Houston, TX USA; 9 Present address: Cleveland Clinic Taussig Cancer Institute, Cleveland, OH USA

**Keywords:** Cancer vaccine, Immune biomarkers, Tumor immunity, Shared antigens, Melanoma

## Abstract

**Background:**

Cancer vaccines are designed to promote systemic antitumor immunity and tumor eradication. Cancer vaccination may be more efficacious in combination with additional interventions that may build on or amplify their effects.

**Methods:**

Based on our previous clinical and in vitro studies, we designed an antigen-engineered DC vaccine trial to promote a polyclonal CD8^+^ and CD4^+^ T cell response against three shared melanoma antigens. The 35 vaccine recipients were then randomized to receive one month of high-dose IFNα or observation.

**Results:**

The resulting clinical outcomes were 2 partial responses, 8 stable disease and 14 progressive disease among patients with measurable disease using RECIST 1.1, and, of 11 surgically treated patients with no evidence of disease (NED), 4 remain NED at a median follow-up of 3 years. The majority of vaccinated patients showed an increase in vaccine antigen-specific CD8^+^ and CD4^+^ T cell responses. The addition of IFNα did not appear to improve immune or clinical responses in this trial. Examination of the DC vaccine profiles showed that IL-12p70 secretion did not correlate with immune or clinical responses. In depth immune biomarker studies support the importance of circulating Treg and MDSC for development of antigen-specific T cell responses, and of circulating CD8^+^ and CD4^+^ T cell subsets in clinical responses.

**Conclusions:**

DC vaccines are a safe and reliable platform for promoting antitumor immunity. This combination with one month of high dose IFNα did not improve outcomes. Immune biomarker analysis in the blood identified several predictive and prognostic biomarkers for further analysis, including MDSC.

**Trial registration:**

NCT01622933.

**Electronic supplementary material:**

The online version of this article (10.1186/s40425-019-0552-x) contains supplementary material, which is available to authorized users.

## Background

Improved approaches for treating metastatic melanoma are needed. The checkpoint blockade antibody therapies (anti-PD-1, anti-CTLA-4) in combination can yield durable responses in up to 58% of patients [1], however, the patients most likely to respond are those who have an existing antitumor response upon which to build [2, 3]. The BRAF and MEK small molecule inhibitors induce dramatic responses of BRAF-mutant melanomas that often endure only months [4]. An area of need is to promote antitumor immunity and tumor immune infiltration where it is lacking. Dendritic cell (DC) vaccines are safe and able to promote antitumor immunity, yet produce durable objective clinical responses in a minority of patients with measurable disease (4.2–7.1%) [5, 6]. Several types of cancer vaccines have shown statistically significant improvement in survival of cancer patients [7, 8] including long-term survival of melanoma patients with DC vaccines [9]. Immunologic vaccine interventions are ideal given their tumor specificity and potential applicability at earlier stages of disease, but new vaccine designs and combinations are needed to improve the induction of an immune response and its durability.

Biomarkers of vaccination efficacy and their correlation with clinical response are areas of active investigation. Many cellular subsets of effector and suppressor cells and clinical lab measures such as absolute lymphocyte count (ALC) have been identified as candidate biomarkers in immunotherapy trials. Promotion of in vivo cross-presentation has been identified by us and others as an important element of vaccine design to optimize clinical outcomes [10–13] and may also be an important mechanism for broadening the tumor antigens responded to [14], potentially including patient-specific mutated antigens, to combat tolerance, host regulatory responses and tumor antigen loss. We and others have found that induction of determinant (or “antigen” or “epitope”) spreading correlates with the development of complete clinical response and improved survival of melanoma [15–21].

In earlier DC vaccine trials, 18 stage III-IV, HLA-A2.1^+^/MART-1^+^ metastatic melanoma patients were treated with autologous immature myeloid DC pulsed with a single HLA-A*0201-restricted epitope (MART-1_27-35_) [15]. The majority of patients were successfully vaccinated, expanding the circulating frequencies of MART-1-specific CD8^+^ T cells. Immunity was not significantly correlated with clinical response. We observed a complete response (CR) in one patient in whom both intramolecular (MHC class I MART-1_27-35_ to MHC class II MART-1_51-73_) and intermolecular (gp100 and tyrosinase) determinant spreading was documented. A related phase II study was conducted, in which 10 stage II-IV melanoma patients received the same MART-1_27-35_ peptide-pulsed autologous DC vaccine. Analysis of these subjects revealed that another patient with a complete clinical response had an immunologic response exhibiting determinant spreading to tyrosinase [16]. In the subsequent trial, in which 14 patients with metastatic melanoma received 3 adenovirus (AdV)-MART-1-engineered DC vaccines (expressing full length MART-1 and inducing MART-1-specific CD8^+^ and CD4^+^ T cells), 3 patients showed evidence of determinant spreading and long overall survival [17]. A better understanding of how to promote effective antitumor immunity with cancer vaccines is critical to further improve outcomes.

Here, we report a novel cancer vaccine clinical trial which was designed to more broadly activate polyclonal antitumor immunity and to promote in vivo cross presentation aiming for improved clinical responses. In this trial, the DC were engineered with 3 full-length defined tumor antigens (tyrosinase, MART-1 and MAGE-A6) to activate multiple CD8^+^ and CD4^+^ T cell clones [22]. Antigen expression driven from the AdV was constitutive, unlike approaches with peptide-based antigen loading [23, 24]. The DC were matured with IFNγ + LPS (as matured DC have been shown to be superior in vivo [25, 26], before AdV transduction, which in vitro has been shown to lead to increased IL-12p70 and superior T cell activation compared to other cocktails [27]. A one-month course of high-dose systemic IFNα-2b (HDI) was tested in one arm of the trial following this vaccine to potentially further boost the immune response. Until 2015, only one adjuvant therapy, IFNα-2b (IFNα), had shown significant benefits in terms of overall and relapse-free survival in multicenter randomized cooperative and intergroup adjuvant trials. The benefits of IFNα have been correlated with induction of autoimmune responses [28–30], and it has been shown to be important in the in vitro polarization and maturation of DC, and the support of memory T cell and NK cell responses [31], promoting full differentiation of naïve cells to CTL [32–36], supporting its testing in this setting.

Together, this personalized DC-based vaccine strategy was devised to more potently activate a polyclonal immune response, incorporating multiple adaptive and innate effectors in order to induce effective anti-tumor immunity and clinical response. We present comprehensive mechanistic immunologic monitoring and biomarker assessments that were prioritized to gain insights into the biology driving clinical response and resistance.

## Materials and methods

### Clinical trial

This was a Phase I, single site study designed to evaluate the toxicity and immunologic and clinical responses from autologous DC transduced with the tyrosinase, MART-1 and MAGE-A6 genes in 30 subjects with recurrent, unresectable stage III or IV melanoma (M1a, b, or c), or resected stage IIIB-C or IV melanoma. The endpoints were 1) local and systemic toxicity, generation of immunity to immunizing antigens and determinant spreading, and clinical response. Enrollment occurred from 9/2012–11/2015, after institutional scientific and IRB approvals (UPCI #09–021) and with informed consent. Most patients were ECOG performance status 0 [17] – 1 [18] and were tested for BRAF and/or NRAS mutation status by sequence analysis (Dept. of Pathology) (Table 1). For most patients, melanoma differentiation antigen staining IHC data from tumors were available (S-100, HMB-45/gp100 and Melan-A were expressed in 27 of 28 tested tumors; tyrosinase, MITF and SOX10 were also tested in 10 cases and were positive) (Additional file 1: Figure S1, Additional file 2: Table S1).

**Table 1:** Patient Demographics and Outcomes

Patient Identifier	Gender	Age	Disease Site	Previous Therapy	Baseline Tumor Mutation^a^	Trial Arm^b^	Best Clinical outcome^c^	Overall Survival^d^	Progression Free Survival^d^
1	F	82	Skin	Temodar 3 cycles (1.5 yrs. prior)	BRAF V 600 K	OBS	PD	6.3	3.2
2	M	74	Skin and chest wall	IL-2, IFNα, ipilimumab	BRAF V600E and NRAS	NR	PD	14.0	2.0
3	F	82	Skin	–	NRAS Q61R	NR	PD	13.6	2.1
4	M	74	Skin	–	None	NR	PD	1.0	0.8
5	M	44	Skin and brain	–	BRAF V600E	NR	PD	0.6	0.4
6	M	67	Nasal cavity	IFNα, vaccine + ipilimumab, IL-2	None	IFN	SD	39.8	5.5
7	M	42	Skin and lymph nodes	Ipilimumab, dacarbazine, IFNα, carboplatin + paclitaxel (2 mo. prior), pembrolizumab	None	OBS	SD	20.0	7.2
8	F	74	Skin	IFNα, DC vaccine, ipilimumab, pembrolizumab (11–090)	None	IFN	SD	13.0	8.7
9	M	66	Skin and lymph nodes	IFN, ipi, anti-PD-1	NRAS Q61K	IFN	SD	27.4	5.7
10	M	61	Skin and retroperitoneum	Ipilimumab	NRAS	OBS	PR	44.3	13.4
11	M	56	Skin and lymph nodes	IFNα, ipilimumab, IL-2, dacarbazine	None	NR	PD	14.8	1.7
12	M	44	Lymph nodes	–	NRAS	OBS	SD	41.7	3.9
13	M	74	Skin	IFNα	None	OBS	NED	42.5	18.4
14	F	58	Skin	IFNα	BRAF V600E	NR	PD	28.4	1.7
15	F	52	Skin	Ipilimumab	NRAS	IFN	NED	42.7	6.9
16	F	75	Vulva	IFNα	None	IFN	NED	38.6	9.3
17	M	64	Lymph nodes	IFNα, ipi, IL-2 + anti-VEGF	BRAF V600E	NR	PD	4.6	2.3
18	M	68	Skin	IFNα	None	OBS	NED	39.9	5.0
19	M	64	Skin	IFNα, DC vaccine	None	OBS	SD	3.5	3.2
20	M	60	Skin	–	None	IFN	PR	40.1	7.3
21	F	61	Skin	IFNα	None	OBS	NED	20.2	19.2
22	F	70	Lymph nodes	Ipi + IFNα	None	OBS	PD	3.0	2.0
23	M	28	Lung	IL-2 + anti-VEGF	None	NR	PD	11.2	2.1
24	F	42	Muscle	Ipi + Nivo, IL-2	None	OBS	PD	3.2	2.1
25	M	52	Skin and lymph nodes	–	BRAF V600K	NR	PD	0.6	0.6
26	M	60	Skin	IFNα	BRAF V600E	OBS	NED	36.1	3.6
27	F	59	Skin	–	n.t.	OBS	NED	35.8	13.7
28	M	47	Lymph nodes	IFNα, Nivo, Ipi	BRAF V600E	NR	PD	11.8	1.6
29	M	60	Skin	–	n.t.	IFN	NED	32.3	14.0
30	F	45	Skin	–	None	IFN	NED	37.5	37.5
31	M	66	Skin	IFNα	None	OBS	NED	37.3	30.7
32	F	41	Skin	IFNα	n.t.	OBS	NED	26.5	2.5
33	F	46	Lower limb and breast	IFNα, Ipi, IL-2, Pembro	BRAF V600E	NR	PD	1.6	0.9
34	M	88	Nasal cavity and lung	IFNα, Ipi, Pembro	None	OBS	SD	8.2	3.3
35	F	52	Skin and lymph node	GM-CSF (14 yrs. prior), IL-2, ipi	BRAF V600E	IFN	SD	24.2	13.0

^a^Tumor mutations identified in available baseline samples tested by clinical pathology sequencing test and/or by NanoString SNV panel, “n.t.” not tested, “None” tested and no mutation detected in panel

^b^Trial arm: *NR* not randomized due to early progression, *IFN* randomized to IFN, *OBS* randomized to observation

^c^*PD* progressive disease, *SD* stable disease, *PR* partial response, *NED* no evidence of disease (all by RECIST)

^d^Time in months

### Treatment

AdVTMM2-transduced DC, (target dose = 10e7, minimum dose = 5 x 10e6, see Additional file 2: Table S1) were given intradermally (i.d.) in the soft tissue adjacent to the axilla or inguinal nodal bed, every two weeks for a total of 3 vaccines. After the administration of the DC vaccines, subjects who had not already progressed (*n* = 23) were randomized to receive either HDI (*n* = 11) or no HDI (*n* = 12). One of the 11 patients randomized 1:1 to IFNα arm refused the treatment after randomization and another patient was not treated due to poor ECOG status. Thus, a total of 9 patients received 1 mo. HDI treatment. Forty-eight patients were screened and 35 patients were enrolled (to offset early progressions) and received at least one vaccine, with 32 of 35 receiving all 3 vaccines.

Subjects randomized to receive the IFNα after DC vaccines received Interferon-α2b (Intron A, Schering-Plough), 20 MU/m2/d (rounded to the nearest 1 million units) administered intravenously for 5 consecutive days (Monday through Friday) weekly for 4 weeks. Administration began 30 days (± 7 days) after the 3rd vaccine. Clinical and laboratory evaluations included the assessment of local and systemic toxicity. Subjects with measurable disease had serial measurement of such target lesions, which were considered for response: at least two unidimensional target lesions were identified for response evaluation. Response was evaluated using RECIST version 1.1.

### DC vaccine preparation

Patients underwent a 3-h leukapheresis procedure at baseline. The total volume of white blood cells collected was 125 mL to 250 mL. The vaccine was prepared according to cGMP guidance in the Immunologic Monitoring and Cellular Products Laboratory (IMCPL). Briefly, the leukapheresis product was elutriated, fractions were tested for % CD45^+^CD14^+/−^ lymphocytes and monocytes, and the fraction with the greatest monocyte purity (31–98%, average = 82%) was plated in DC medium (Cell Genix) with 1000 U/mL GM-CSF (Genzyme and Sanofi) and 1000 U/mL IL-4 (Cell Genix). Cytokines were replenished day (d) 3 or 4, and on d5 the immature DC (iDC) were matured with 1000 U/mL IFNγ (Actimmune and R&D Systems) + 250 ng/mL LPS (Sigma-Aldrich) overnight. The matured DC (mDC) were harvested 18–24 h later, and 4.5 x 10e7 were transduced with AdVTMM2 [22] at MOI = 400. These AdVTMM2/DC underwent safety (sterility, mycoplasma, endotoxin) and identity release testing. DC identity was confirmed by flow cytometry. DC were large, granular lymphocytes at ≥70% purity (range = 92–100%, average = 97%) that expressed CD11c (92–100%, avg. = 98%), HLA-DR (92–100%, avg. = 98%), CD40 (92–100%, avg. = 98%), CD86 (88–100%, avg. = 96%), CD80 (17–99%, avg. = 90%). For maturation, CD83 (13–97%, avg. = 68%) and CCR7 (8–70%, avg. = 22%) were also measured, but were not used as release criteria. The first vaccine was administered fresh, and the cells for V2 and V3 were cryopreserved in 10% DMSO/40% AB serum/50% Cell Genix medium, thawed and reformulated for injection. Fresh and thawed vaccines were shown to be viable and phenotypically stable for at least 4 h at 4 °C. Fresh DC were cultured as previously described [37] for 24 h and supernatants frozen for batch testing of IL-12p70 and IL-10 secretion by Luminex. For molecular analysis, 5x10e6 iDC, mDC and vaccine DC were each resuspended in RNAlater (Qiagen), RNA was isolated and tested on HUGENE 2.0 ST arrays (Affymetrix) Additional file 1: Figure S1.

### Clinical laboratory testing

Blood was drawn for clinical lab assessments of blood cell counts, anti-nuclear antibodies (ANA), thyroid stimulating hormone (TSH) and rheumatoid factor (RF), as well as lactate dehydrogenase (LDH) and C-reactive protein (CRP). Several of these clinical lab biomarkers were previously correlated with outcome of melanoma in prior trials of immunotherapy [38–42]. Autoimmune toxicities were assessed by ANA, TSH and RF assays, detection of other autoimmunity symptoms and visual observation of vitiligo.

### Immunologic monitoring

Cells from the baseline leukapheresis and the additional 90 min. d43 (post DC vaccines) and d89–101 (post observation of IFNα, referred to as “d89” for simplicity) leukaphereses were used for most assays. If a subject refused or the second or third leukapheresis was not able to be performed, 140 mL of blood was drawn (green top heparin tubes) as an alternative (pt. 12 at d89, pt. 15 at d89, pt. 18 at d89, pt. 24 at d43, pt. 31 at d89). In addition, one heparinized tube of whole blood was drawn at each time point from each patient (*n* = 35) for fresh whole blood flow cytometry to obtain absolute counts and percentages of PBMC subsets (Beckman TQ-prep, Beckman Coulter). Healthy donor (HD) controls were obtained (with informed consent, under UPCI #04–001 protocol) to use as assay controls and as comparators for melanoma patients results (n = 35 from 27 different individuals). Cells were stained for CD3, CD4, CD8, CXCR3, CCR6, CCR7, CD45RA, CD25, CD127, FOXP3, CD14, CD15, CD16, CD11b, CD33, CD56, CD69, HLA-DR, NKG2D (CD314) and lineage (CD3, CD19, CD56). Remaining PBMC were isolated via Ficoll and cryopreserved. Flow cytometry was performed on a Coulter FC 500 after daily QC with cytometer set up and tracking beads and analyzed with CXP software. The IMCPL is CAP accredited for flow cytometry and has participated in flow cytometry gating proficiency panels.

A red top tube (no anticoagulant) was also drawn at each time point for serum to test for circulating antibody and cytokine/chemokine/growth factor levels. Serum was clotted at room temperature, aliquoted and frozen at − 80 °C. Serum was kept in a monitored freezer and tested after a single thaw. All procedures were performed in the IMCPL by competency-trained technologists according to SOP’s in a CAP/CLIA laboratory environment.

### Direct IFNγ ELISPOT assays

Antigen specific T cell responses were examined by two standardized IFNγ ELISPOT assays. An assay with total PBMC was performed at baseline and d43, testing responses to autologous DC transduced with individual antigens AdVtyrosinase, AdVMART1, AdVMAGEA6, AdVLacZ (for the AdV vector backbone response) and controls (*n* = 31 patients). A second assay was run after d89 with magnetic bead purified CD8^+^ and CD4^+^ (Miltenyi) T cells as responders against autologous DC transduced with individual antigens or pulsed with synthetic peptides and controls (*n* = 28). The results of the first PBMC ELISPOT were 70% concordant with the subsequent, more detailed ELISPOT assay testing purified CD8^+^ and CD4^+^ T cells (despite the different methods), and the second assay identified more T cell responses than the total PBMC assay. Wells were plated in triplicate and a healthy donor control included on each plate served as an assay and reagent control. Plates were analyzed on a CTL Technologies ImmunoSpot reader (Shaker Heights, Ohio). To statistically determine responses to the melanoma associated antigens, the AdVLacZ response was subtracted from the AdV-melanoma antigen response. For peptide groups, the T2 only response was subtracted from the T2+ peptide response. The AdV response was considered the AdVLacZ response and the (T2 + hexon peptide)-T2 only response. A positive increase was considered at least 10 spots counted and a two-fold or greater net spot increase. The IMCPL participates in external ELISPOT proficiency panels.

### Serum testing

Multiplexed Luminex 45-plex (Affymetrix/Thermo-Fisher ProcartaPlex) testing of sera was performed to profile the serum samples and to examine a previously identified baseline “pro-inflammatory cytokine signature” [43] of IFNα response in melanoma patients (IL-1α, IL-1β, IL-6, TNFα, MIP-1α and MIP-1β), and test additional immune mediators for correlation with clinical and immune outcomes, according to manufacturer instructions. The following analytes were included in this analysis: BDNF; Eotaxin/CCL11; EGF; FGF-2; GM-CSF; GRO alpha/CXCL1; HGF; NGF beta; LIF; IFN alpha; IFN gamma; IL-1 beta; IL-1 alpha; IL-1RA; IL-2; IL-4; IL-5; IL-6; IL-7; IL-8/CXCL8; IL-9; IL-10; IL-12 p70; IL-13; IL-15; IL-17A; IL-18; IL-21; IL-22; IL-23; IL-27; IL-31; IP-10/CXCL10; MCP-1/CCL2; MIP-1 alpha/CCL3; MIP-1 beta/CCL4; RANTES/CCL5; SDF-1 alpha/CXCL12; TNF alpha; TNF beta/LTA; PDGF-BB; PLGF; SCF; VEGF-A; VEGF-D. Healthy donor sera served as additional controls. In addition, the Affymetrix/Thermo-Fisher Procarta Plex Human Checkpoint 14-plex was utilized, which included detection of the soluble forms of: BTLA, CD137/41BB, CD152/CTLA-4, CD27, CD28, CD80, GITR, HVEM, IDO, LAG-3, PD-L1, PD-L2, PD-1 and TIM-3. The IMCPL participates in external Luminex proficiency panels run by the NIAID EQAPOL program.

### Biostatistics and bioinformatics analyses

Originally, 15 patients/arm were planned for the trial. To estimate power, we assume that data are normally and use the simplification that only one kind of assay is performed. With a one-sided level 0.05 test and 15 subjects per arm, there is 80% power to detect an effect size of 0.96, where effect size is the difference in group means divided by the within-group standard deviation (δ/σ). However, the target accrual was not fully reached due to several early progressors who did not complete post-DC vaccine treatment and exhaustion of funds.

All correlative data analysis was conducted using R (version 3.4.3) and Bioconductor. An *p*-value of 0.05 or less was considered statistically significant. No adjustments for multiple comparisons were performed. Kaplan-Meier analysis was used to estimate the median OS and PFS. Logrank tests were used to compare the OS and PFS between different study arms. Serum cytokines with detection rate < 50% were dichotomized, and those with > = 50% detection rate were treated as continuous endpoints. Serum cytokine level before (d43) and after (d89) the HDI treatment were compared using McNemar’s tests (for dichotomize cytokines) and Wilcoxon Signed-Rank tests (for continuous cytokines). Lasso Cox proportional hazard (PH) models were used to select a panel of cytokines at baseline, d43 and d89 that are associated with OS. The selected cytokines were then fit into a Cox PH model to assess the association of each marker and OS. Correlation of ELISPOT assays with clinical outcome was determined by performing a student’s t test of the larger of the magnitude of the responses from days 43 and 89 (see above) for responders, defined as patients with PR or SD, against non-responders.

Vaccine Phenotype and Cytokine Production Analysis: Correlation of DC cell surface markers (e.g., CD83) and secretion levels of IL-12p70 and IL-10 with vaccine antigen response was determined by performing a student’s t-test of the expression level for patients who were vaccine antigen responders against those who were non-responders.

Circulating T Cell Frequencies and Clinical Blood tests: Analysis of flow cytometry assessing the circulating frequencies of naïve T-cells, T-regulatory cells, MDSCs, NK-cells, DCs, and chemokine receptors, and of lymphocyte, neutrophil, monocyte, eosinophil, and basophil counts were processed to compare absolute and percentage cell counts at baseline, d43 and d89. Change between time points was determined by calculating both the difference and fold change between the time points. Statistical testing of counts was determined using a student’s t-test.

Serum Anti-AdV Neutralizing Antibody Titers: Antibody titers at 1:4 and 1:8 dilutions as measured by MFI were analyzed by using a student’s t-test for responders and non-responders.

Serum Cytokine Assessments: Analysis of serum cytokine data was performed to compare levels of cytokines, chemokines, growth factors, and soluble checkpoint proteins at baseline, d43, and d89. Change between time points was determined by calculating both the difference and fold change between the time points. Statistical testing was performed using a student’s t-test.

DC vaccine microarrays: analysis was performed using the Robust Multi-Average (RMA) method.

## Results

### Patients, demographics and toxicities

Patient demographics are shown in Table 1, and inclusion/exclusion criteria are detailed in Additional file 2: Table S2. At enrollment, 3 patients were stage IIb/IIc, 10 were stage III, and 18 were stage IV (4 unknown). The average age was 60 (range 28–88). To date, DC vaccines have generally shown little toxicity, while IFNα has a more significant toxicity profile. Of 35 evaluable, 18 patients experienced grade 1–4 toxicity relevant to the treatment. Nine were grade (G)1 toxicities attributed to the DC vaccine injection while the other 9 (2 x G2, 6 x G3, 1 x G4) were attributed to IFNα (Additional file 2: Table S3). Because autoimmune phenomena such as the development of vitiligo may indicate a broadening of antigen reactivity and correlate with determinant spreading, an array of clinical laboratory autoimmune tests were performed. Several patients had elevated baseline ANA (pt. 24, 30), RF (pt. 5, 16, 34) or TSH (pt. 7, 16, 21, 22, 30) values. After receipt of the DC vaccines, the ANA values of 3 patients (pt. 26, 28, 35), RF value of 1 (pt. 19) and TSH of 1 (pt. 35) increased to above normal. None of the patients receiving one month of IFNα showed values that changed for these tests or manifested any other signs of autoimmunity. Vitiligo was not observed.

### Clinical outcomes

**Fig. 1:**
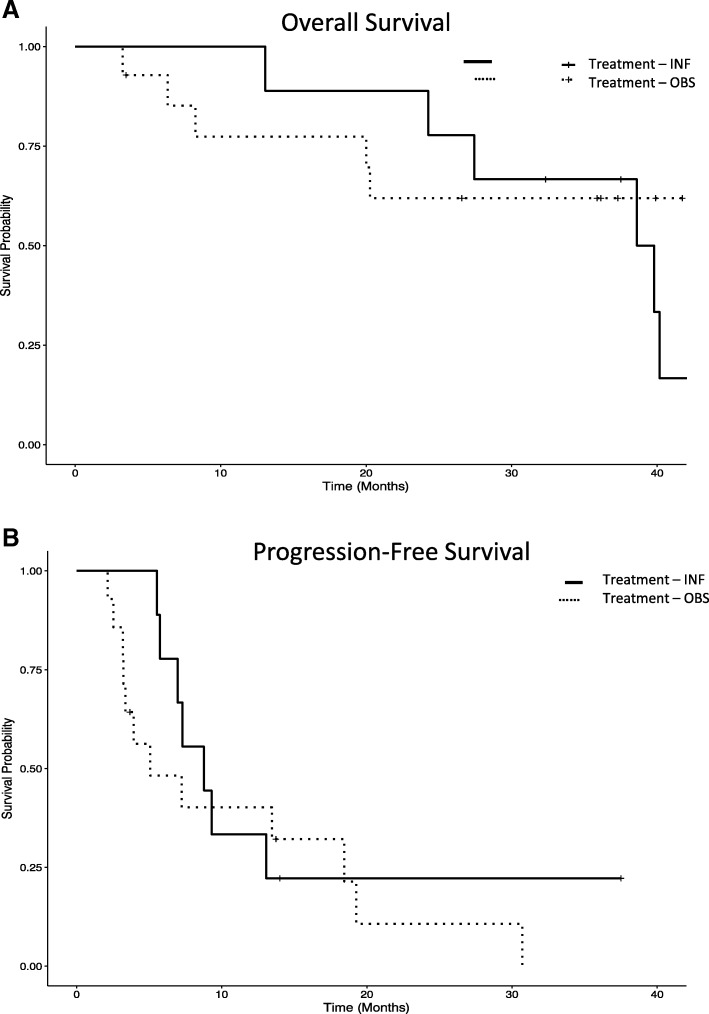
Kaplan-Meier plot of OS and PFS. **a** and **b**. OS and PFS is shown for patients who were randomized after DC vaccines (*n* = 23) to observation (no boost, *n* = 12 randomized + 2 not receiving HDI) or those randomized to HDI (*n* = 11 randomized, 9 receiving IFN). OS: IFN vs. OBS *p* = 0.54 (ns). PFS: IFN vs. OBS *p* = 0.43 (ns). Not shown are those who progressed early, before randomization (n = 12), who were the statistically significant different clinical group vs. those randomized (OS *p* = 0.0001, PFS *p* < 0.0001)

Thirty five patients were evaluable, 14 patients are still alive (40%) and 21 (60%) have died. Median overall survival (OS) is 36 months, and median progression free survival (PFS) is 17.3 months (Fig. 1). Clinical responses include: 14 progressive disease (PD) (13 PD before randomization), 2 partial responses (PR, 8.33% of measurable disease patients), 8 stable disease (SD, 3.2–13.1 months) among the 24 patients with measurable disease. Among the 11 patients with no evidence of disease (NED) at baseline, 7 have since recurred, 4 remain NED (3.7–37.5+ months). The Kaplan-Meier plots of OS and of PFS are shown in Fig. 1. There is a significant difference between those patients that progressed during or immediately after the DC vaccines and were not able to be randomized, compared to those who were randomized to receive the IFNα boost or observation. There was no significant difference detected between the OBS and IFN groups for OS or PFS.

### DC vaccine dose, phenotype and cytokine production

Of the 35 patients enrolled, 31 received the full 10^7^ DC target dose in the first fresh vaccine, with an overall average of 9.55 × 10^6^ DC in the first vaccine (Additional file 2: Table S1), and an average of 7.29 × 10^6^ thawed DC were delivered in V2 and V3. There were no correlations suggesting a dose-response effect on clinical outcome in this range of DC numbers, which is in agreement with previous trials [15, 44–46].

The DC phenotype and the IL-12p70 and IL-10 cytokine secretion upon CD40 ligation (Additional file 2: Table S1) were measured for each vaccine batch. The DC phenotype was tested as an identity measure, as variation in the levels of these surface proteins has not generally correlated with immune or clinical outcome. One of the most variable measures, CD83 (DC maturation related) inversely correlated with induction of a vaccine antigen-specific T cell response (lower CD83% positive (*p* = 0.0050) and lower CD83 MFI (0.054)), which was unexpected. While all DC vaccines were strongly positive for MHC class I and II and costimulatory molecules, lower HLA-DR MFI (*p* = 0.041) and CD80 MFI (0.079) were each similarly associated with superior T cell response induction. IL-12p70 secretion was a particular focus to determine if higher CD40L-induced production would correlate with improved clinical outcome, as we and others have shown previously [44]. Unexpectedly, the two PR patients’ DC vaccines produced lower IL-12p70 (348 and 124 pg/mL, below the overall average of 875 pg/mL and well below the highest value of 7177 pg/mL) than many with PD. The secreted IL-10 levels were also determined (73 and 197 pg/mL produced by the PR patient DC vaccines respectively (overall average 432 pg/mL). These data suggest that standard DC phenotyping, IL-12p70 and IL-10 secretion levels, were not of value as DC potency assessments in this study. Analysis of RNA microarray data from iDC and vaccine cells suggest that the suppressive molecular indolamine 2,3-dioxygenase-1 is upregulated in vaccine DC and that several metabolic genes are significantly downregulated in vaccines from patients with good (PR + SD) clinical outcomes suggesting pathways for future interrogation (Additional file 1: Figure S2).

### Functional T cell responses

**Fig. 2:**
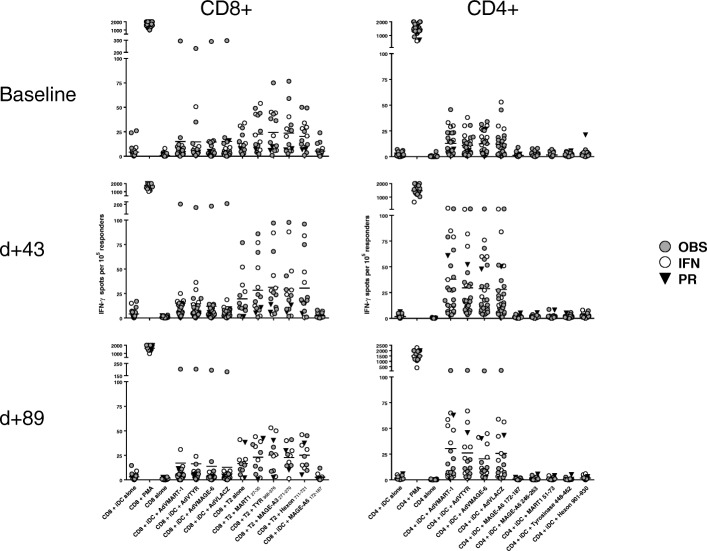
IFNγ ELISPOT assay for purified CD8^+^ and CD4^+^ T cells. A direct ELISPOT was performed to determine the frequency of T cells specific to full length antigens expressed in autologous DC and previously characterized peptides (*n* = 28 patients). Assay controls (no antigen, PMA + ionomycin) are also shown. The circle symbols denote trial arm, and the responses of the two PR patients are also noted (filled triangle)

The primary goal of this DC vaccine was to induce polyclonal CD8^+^ and CD4^+^ T cell immunity to the three melanoma-associated antigens encoded by the AdV. Two different ex vivo ELISPOT assays were performed. The first tested total PBMC for responses to each full-length antigen in autologous DC, directly after the 3rd DC vaccine (d43) which served to keep the two trial arms balanced for T cell response (data not shown). The second ELISPOT was performed after IFNα or observation at d89, and tested separated CD4^+^ and CD8^+^ T cells, and also included HLA-A2-restricted and MHC class II restricted peptides (Fig. 2). These data show that, at baseline, 10 of 31 patients tested already manifest melanoma antigen specific T cell responses to tyrosinase, MART-1 and/or MAGE-A6 and 16 of 31 had recall responses to AdV antigens. After the DC vaccines, 18 of 31 (58%) tested had developed increased T cell responses over baseline to vaccine-encoded melanoma antigens (Tyrosinase: 7 CD8^+^/4 CD4^+^; MART-1: 6 CD8^+^/7 CD4^+^; MAGE-A6: 4 CD8^+^/ 2 CD4^+^ responses across 18 pt.). Of the 13 patients without a vaccine antigen response, 6 had increased AdV T cell responses, indicating successful vaccination and an AdV-specific T cell response. Fifteen patients (of 31 tested) were positive at baseline for AdV T cell response (12 CD4^+^/7 CD8^+^, and the increases noted among 16 pts. were more commonly in the CD4^+^ T cell compartment (14 pt. vs. 5 with CD8^+^ T cell response increases). It was hypothesized that AdV-specific T cell responses might induce increased melanoma antigen-specific T cell responses [47]. On the contrary, there were more CD8^+^ vaccine antigen-specific responses in patients without AdV-specific T cell responses. Both patients with PRs had increased IFNγ-producing T cells to the vaccine-encoded antigens, as well as some of the patients with NED, SD and PD clinical responses. The magnitude of the vaccine antigen T cell response did not correlate with clinical outcome.

### Circulating T cell frequencies

Fresh peripheral blood was examined at each time point to examine changes in effector, suppressor and antigen presenting cells. By testing whole blood, single platform data on frequencies and absolute percentages were obtained [48]. CD4^+^ and CD8^+^ T cells were tested for effector/memory phenotype and chemokine receptor expression. The DC vaccines could have a systemic impact on these cells and their levels at baseline may impact the ability to respond to the vaccine.

**Fig. 3:**
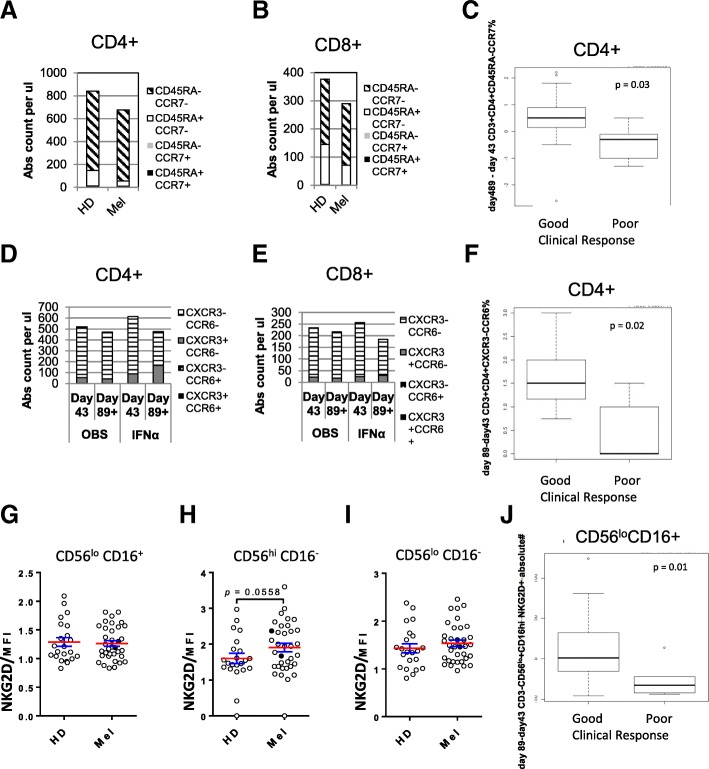
Whole blood T and NK cell phenotyping. **a**-**f** The percentages and absolute counts for CD3^+^CD4^+^ and CD3^+^CD8^+^ T cells expressing naïve/effector/memory markers (CD45RA, CCR7) (**a**-**c**) or trafficking markers (CXCR3, CCR6) (**d**-**f**) are shown in melanoma patients (*n* = 35 patients) in comparison to HD controls (n = 35) (**a**, **b**) or by trial arm (**d**, **e**). Box plots for significant correlations with clinical response are shown (**c**, **f**, **j**). **g**-**i** NK cell subset phenotyping for NKG2D expression levels as shown for HD and patients

Examination of CD4+ and CD8+ T cells show that at baseline, patients have reduced CD4+ and CD8+ T cell counts, as well as reduced frequencies of absolute effector T cell (Teff) phenotype cells (CD45RA^−^CCR7^−^) compared to HD (Fig. 3a and b). After DC vaccines, the frequencies of circulating Teff were further reduced (not shown). IFNα led to a small decrease in the frequency of absolute numbers of CD4^+^ and CD8^+^ Teff (not shown).

At multiple timepoints, several characteristics of circulating CD4^+^ and CD8^+^ T cells showed significant correlations with good clinical response: increases at d43 of CD3^+^CD8^+^CD45RA^+^CCR7^−^ absolute counts, and increases in % CD3^+^CD4^+^CD45RA^−^CCR7^+^ at d43 and at d89 (Fig. 3c). Baseline measures of CD3^+^CD8^+^CD45RA^+^CCR7^−^ and CD3^+^CD4^+^CD45RA^−^CCR7^−^ absolute counts correlated with clinical outcome (fewer CD8+ and more CD4+ with good outcome) (not shown). These data suggest that CD8^+^CD45RA^+^CCR7^−^ T cells, which may represent peripheral trafficking of naïve or CD8^+^ T cells, and CD4^+^CD45RA^−^ cells are of particular interest.

T cell trafficking was further examined with CCR6 (MIP-3α receptor) and CXCR3 (IP-10/MIG/ITAC receptor) associated with intratumoral trafficking in melanoma [49]. Both the percent and absolute numbers of CD4^+^CXCR3^−^CCR6^−^ T cells decreased between d43 and d89 (Fig. 3d) across the patient groups. IFNα increased the circulating CXCR3^+^CCR6^−^CD4^+^ T cells (Fig. 3d). A similar but more modest increase was seen in CXCR3^+^ CD8^+^ T cells (Fig. 3e). Correlations of chemokine receptors on CD4^+^ and CD8^+^ T cells with clinical response showed that increases in CXCR3^−^ cells were correlated to good clinical outcome (Fig. 3f). Correlations between Treg, MDSC and other subpopulations with clinical outcome were minimal.

### Circulating NK cell frequencies

The circulating frequencies of three NK cell subsets (CD3^−^CD56^high^CD16^−^, CD3^−^CD56^low^CD16^+^ and CD3^−^CD56^low^CD16^−^) were tested, as well as their activation state (CD69, NKG2D). At baseline, melanoma patients had slightly reduced absolute counts of NK cells compared to HD and after DC vaccinations, frequencies were not significantly changed (L. Vujanovic, 2018 submitted). NK cells expressing NKG2D were slightly increased at baseline on CD56^high^/CD16^−^ cells in melanoma patients, while on CD56^low^CD16^+^ cells NKG2D was slightly reduced after DC vaccination (Fig. 3g,h, and i). Several significant clinical correlations of early (baseline – d43) and late (d43- d89) changes in NK cell levels were identified. An increase in CD3^−^CD56^+^ and CD56^low^CD16^+^NKG2D^+^ absolute counts correlated with good clinical outcome (*p* = 0.02 and 0.01, respectively). CD56^low^CD16^+^ absolute counts, percentage and CD56^low^CD16^+^NKG2D^+^ absolute counts were also positive correlated with good outcome (*p* = 0.006, 0.005 and 0.018 (Fig. 3j), respectively), suggesting an important role for the cytotoxic CD56^low^CD16^+^ NK cell subset.

### Treg and MDSC

**Fig. 4:**
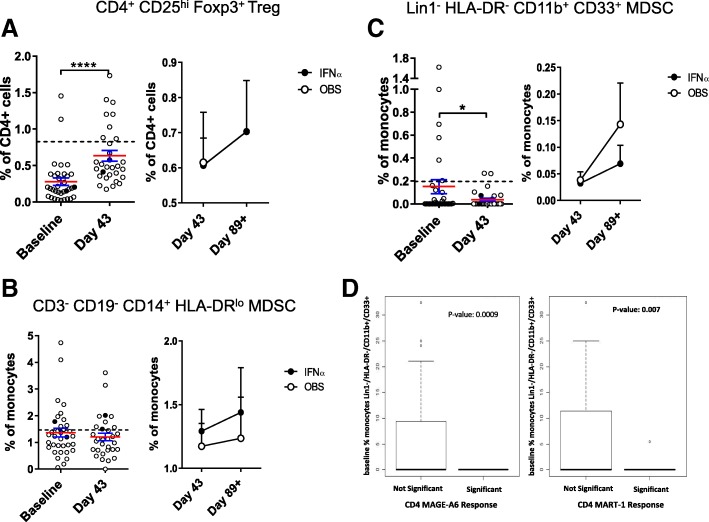
Circulating suppressor cell frequencies. The frequencies of (**a**) Treg and subsets of myeloid (**b, c**) MDSC are shown (n = 35 patients). The left side of each panel shows the difference between baseline and d = 43 post DC vaccines. The right side of each panel shows the change between d43 and d89 for each trial arm. Dotted lines represent median HD values (n = 35). Two examples of significant correlations between MDSC frequencies and patient development of vaccine antigen-specific T cell responses are shown (**d**)

Frequencies of suppressive Treg and MDSC were also tested. Treg were detected in patient blood at baseline and increased after DC vaccination (Fig. 4a). IFNα did not impact the Treg frequencies. Gating based on CD127^low^ instead of intracellular FOXP3 showed similar results (data not shown). Three subsets of MDSC were tested [50]. The DC vaccines resulted in a decrease in rare circulating HLA-DR^−^CD11b^+^CD33^+^ MDSC, but not in the higher frequency HLA-DR^low^/CD14^+^ MDSC (Fig. 4b, c). Similarly, CD15^+^ gMDSC were not impacted by the DC vaccines (not shown). IFNα had a modest impact on different MDSC subsets (Fig. 4 and not shown). Notably, it was MDSC whole blood phenotyping that correlated significantly with induction of vaccine antigen-specific T cell responses (22 of 44 significant measures). Lower MDSC frequencies, decreases over time and lower baseline MDSC values were all correlated to positive CD8+ and CD4+ T cell responses (Fig. 4d and not shown). Treg correlations were mixed, with both higher and lower Treg frequencies correlating with increased T cell responses (similar to previous findings [51–53]).

### Circulating NKT cells

Interestingly, baseline percentages of CD3^+^CD56^+^ NKT cells in whole blood were significantly correlated with improved clinical outcome (*p* = 0.010). A late decrease (d43 to d89) was significantly correlated to good outcome (*p* = 0.02). To investigate this further, banked lymphocytes and DC were tested for CD3^+^/CD1d tetramer + invariant NKT cell frequency and function. Several patients were tested (2 PR, 2 SD, 3 NED and 3 PD), compared to healthy donors. The NKT cell frequencies were very low (0.0019–0. 0393%). Three of 8 patients showed a > 2x increase in NKT cell frequency at d43 after DC vaccines (2 PR and 1 PD), however activation and cytokine production were not notably changed after the vaccines (data not shown).

### Clinical blood cell assessments

Patients also had absolute lymphocyte, neutrophil, monocyte, eosinophil and basophil counts tested. Several patients had abnormal counts at baseline. Analysis showed a trend towards an early increase in neutrophils (baseline to d43, *p* = 0.066) correlating with poor clinical outcome, and a late decrease in monocytes (*p* = 0.014), correlating with vaccine-antigen-specific T cell response.

### Serum anti-AdV neutralizing antibody titers

Serum was examined for circulating anti-AdV neutralizing antibody (Nab) responses. As expected, the patients had differing titers of Nabs at baseline. After AdVTMM2/DC vaccination (d43), most patients had a strong increase in Nab titers (Additional file 1: Figure 3a, b). In most cases, these titers were reduced by d89/101. We hypothesized that the baseline presence of Nabs or vaccine-induced increases in Nab levels would negatively impact the ability to develop a T cell response to the vaccine (by IFNγ ELISPOT). No significant correlations were identified with immune response or clinical outcome, which is in agreement with an earlier study in a murine model with pre-immunization with AdV before AdV/DC vaccination [51].

### Serum cytokine assessments

Serum was examined for cytokines, chemokines, growth factors and soluble checkpoint proteins that could describe the immune milieu of the patients through the trial and show possible changes occurring with the DC vaccines or IFNα. Correlations with most analytes were not significant. Higher IL-7 at d89 was correlated with positive vaccine antigen T cell response (*p* = 0.007). Superior clinical response was correlated with higher d43 IL-18, LIF and RANTES (all *p* < 0.05) (not shown). Serum cytokine level before (d43) and after (d89) the HDI treatment were compared in the 9 patients received HDI treatment. IP-10 and VEGFα were found to be significantly increased post HDI treatment (*p* values = 0.03 and 0.01 respectively). We also specifically examined a previously identified pro-inflammatory signature associated with survival after IFNα [43]. Among the 6 cytokines that were found to be significantly higher in the serum of patients with longer RFS in our previous study, IL-1α, IL-1β and IL-6 were not detected in the serum samples. TNF-α, MIP-α and MIP-β were found to be higher in pre-treatment samples (d43) of patients with longer OS, which is consistent with the previous finding, however, none reached statistical significance. We further selected a combination of serum cytokine prognostic biomarker at baseline and found that VEGF A and D are significantly negatively associated with OS (*p*-values = 0.0001 and 0.02 respectively) in patients with measurable disease (*n* = 24).

The levels of soluble checkpoint and costimulatory molecules were of particular interest, given recent studies indicating the clinical impact of targeting these cell-associated proteins [54, 55], and the previous treatment of some trial patients with CTLA-4 or PD-1 blocking antibodies (Table 1). At baseline, patients with good clinical outcome had a trend towards lower serum TIM-3 (*p* = 0.07), while higher soluble CD27 at d43 was also correlated with superior clinical response (*p* = 0.05).

## Discussion

This trial was designed with two major goals: [1] to prospectively test the efficacy of a new DC based AdV engineered vaccine and [2] to evaluate the subsequent role of one month of HDI following the DC vaccination, to determine its effect in a randomized prospective trial setting.

To date, the role of the HDI adjuvant therapeutic regimen has not been carefully examined in a prospective setting for its ability to augment therapeutically relevant type 1 responses to melanoma-associated antigens. The published literature includes only one report of the ad hoc application of this regimen following a canarypox-based genetic vaccination regimen (ALVAC, [56]), in which 4 of 7 patients demonstrated significant augmentation of immune response to the gp100 vaccine, of which two patients with measurable tumor exhibited objective antitumor responses that correlated with development of increased levels of CTL directed at gp100 positive tumor cells. Our results indicate that [1] the DC based AdV vaccine is immunogenic in the population of advanced and multiply treated patients tested here (including after checkpoint blockade), and [2] that the addition of HDI to the DC vaccine did not augment either vaccine antigen-specific T cell frequencies detected, nor the clinical responses induced by the DC vaccine. Other vaccine combinations are currently in clinical testing by several groups, including with checkpoint blockade.

The identification of DC vaccine potency measures addressed in this trial remains a pivotal one for the field. Phenotypic measures are important cell identity tests, but to date, levels of specific molecules or maturation levels (CD83, CCR7) have not correlated well with induced T cell responses or clinical outcomes. In this data set, it was somewhat lower levels of several antigen presentation and costimulatory molecules that showed superior immune response, although all were highly expressed on the DC surface. These data show that standard DC phenotype measures are not useful potency measures. We also measured both spontaneous and CD40L-induced IL-12p70 and IL-10 production by the DC vaccines. The IL-12 secretion levels were not significantly correlated with improved T cell response or clinical outcome, unlike some previous trials [57] and in vitro data [32]. On the contrary, in this study, there was a trend for lower IL-12p70 and positive clinical response. Together, these data demonstrate that more high-throughput DC vaccine profiling approaches are needed to identify critical potency parameters that will allow for needed progress in the DC vaccine field (Additional file 1: Figure S2, and Maurer, Stroncek, Butterfield et al., in preparation).

Adenovirus is a gene delivery vehicle with a long safety record. The rationale for AdV mediated antigen transgene expression included efficient antigen gene transfer into human DC [23, 27], long-term transgene expression [58], maturation effects [24, 27], superior T cell activation when compared to peptide-pulsed DC [59–62], activation of CD8^+^ and CD4^+^ T cells simultaneously [59] and a unique profile of chemokine secretion and NK cell migration [24, 63, 64]. While pre-existing Nabs to this vector from prior environmental exposure was a concern, we previously demonstrated in mice pre-immunized with AdV that subsequent immunization with antigen-engineered DC yields the same high level of immunity. [65, 66]. In this study, we identified T cell responses to the AdV which were generally CD4^+^ T cell responses and detected an increase in Nabs in most patients, regardless of baseline levels. Because AdV-specific T cells are robust type I cells [47, 67], we hypothesize that their activation in the milieu of tumor antigen presentation may support tumor antigen-specific T cells. The AdV-specific T cell responses did not detectably impact vaccine antigen T cell or clinical responses. While the magnitude of anti-AdV nAbs was not expected, the titers did not correlate with T cell or clinical response.

To examine circulating effector and suppressor cells (without any potential bias or loss due to Ficoll isolation or cryopreservation), we tested whole blood directly by flow cytometry. A number of patterns emerged. Clinical response was significantly correlated to several measures of CD8^+^ and CD4^+^ effector and effector memory T cells (which may correspond to some subsets previously examined [68, 69]. Additional profiling of these T cells is underway (Santos, in preparation). In contrast, the functional T cell response to vaccine antigens by ELISPOT assay significantly correlated with the circulating frequencies of Treg and MDSC, indicating that these suppressive cell types were playing a key role in modulating the development of melanoma antigen T cell responses. As in our previous studies in melanoma [51–53], both higher and lower frequencies of Treg correlated with improved immune outcomes. MDSC were a more robust negative correlate of immune response as we and others have reported, suggesting a more prominent role in antitumor immunity [52, 53, 70, 71].

While our preclinical data demonstrating a potent impact of the AdV/DC vaccine on NK cell activation was not recapitulated in vivo, the NK analysis did yield several novel observations. We revealed significant increases in baseline NKG2D in melanoma patient CD56^hi^/CD16^−^ NK cells, and correlations of CD56^low^/CD16^+^ that are currently being further investigated in depth [72]. The serum profiling performed included assessment of soluble checkpoint and costimulatory molecules. Significant correlations with IL-7, IP-10, VEGF, soluble CD27 and soluble TIM-3 with clinical and T cell responses were identified.

## Conclusions

We performed a genetically engineered DC vaccine trial that induced polyclonal CD8+ and CD4+ antigen-specific T cell responses, as well as tumor regression or stabilization in several subjects. These effects were not improved by one month of HDI. Our DC vaccine potency testing and immune biomarker profiling identified several significant baseline and on-treatment cellular subsets and circulating soluble proteins that impact both immunologic and clinical outcomes and aid the development of more effective cancer vaccines.

## Additional files

Additional file 1: **Figure S1.** DC vaccines were phenotype for cell surface protein expression as described in the Materials and Methods. The bar graphs show relative expression across all DC vaccines for comparison. Markers are color coded as shown in the legend. **Figure S2.** Patient DC vaccine microarray data was normalized using the Robust Multi-Average (RMA) method. Differential expression analysis was performed on the normalized microarray data to determine significant genes by A) Clinical Response: (PR, SD) VS (NED, PD) and in B) AdVTMM2 DC, compared to immature DC. The Benjamini and Hochberg test was applied to control for False Discovery Rate and the p-value cutoff was set to 0.05. RMA normalization was performed using the Oligo package in R. **Figure S3.** Differential Expression analysis was performed using the Limma package in R. (PPTX 14100 kb)

Additional file 2: **Table S1.** DC vaccine doses and cytokine production. **Table S2.** Inclusion and Exclusion Criteria. **Table S3A.** Worst Grade AE per Patient. **Table S3B.** Adverse Events of Grade 3 or 4. (DOCX 90 kb)
