# Supplementary material for: Multiple antigen-engineered DC vaccines with or without IFNα to promote antitumor immunity in melanoma
Source: J Immunother Cancer. 2019 Apr 24;7:113. doi: 10.1186/s40425-019-0552-x (PMC6480917; doi:10.1186/s40425-019-0552-x)
Supplement: Supplementary file 1 — Figure S1. DC vaccines were phenotype for cell surface protein expression as described in the Materials and Methods. The bar graphs show relative expression across all DC vaccines for comparison. Markers are color coded as shown in the legend. Figure S2. Patient DC vaccine microarray data was normalized using the Robust Multi-Average (RMA) method. Differential expression analysis was performed on the normalized microarray data to determine significant genes by A) Clinical Response: (PR, SD) VS (NED, PD) and in B) AdVTMM2 DC, compared to immature DC. The Benjamini and Hochberg test was applied to control for False Discovery Rate and the p-value cutoff was set to 0.05. RMA normalization was performed using the Oligo package in R. Figure S3. Differential Expression analysis was performed using the Limma package in R. (PPTX 14100 kb) [file 40425_2019_552_MOESM1_ESM.pptx]

## Slide 1
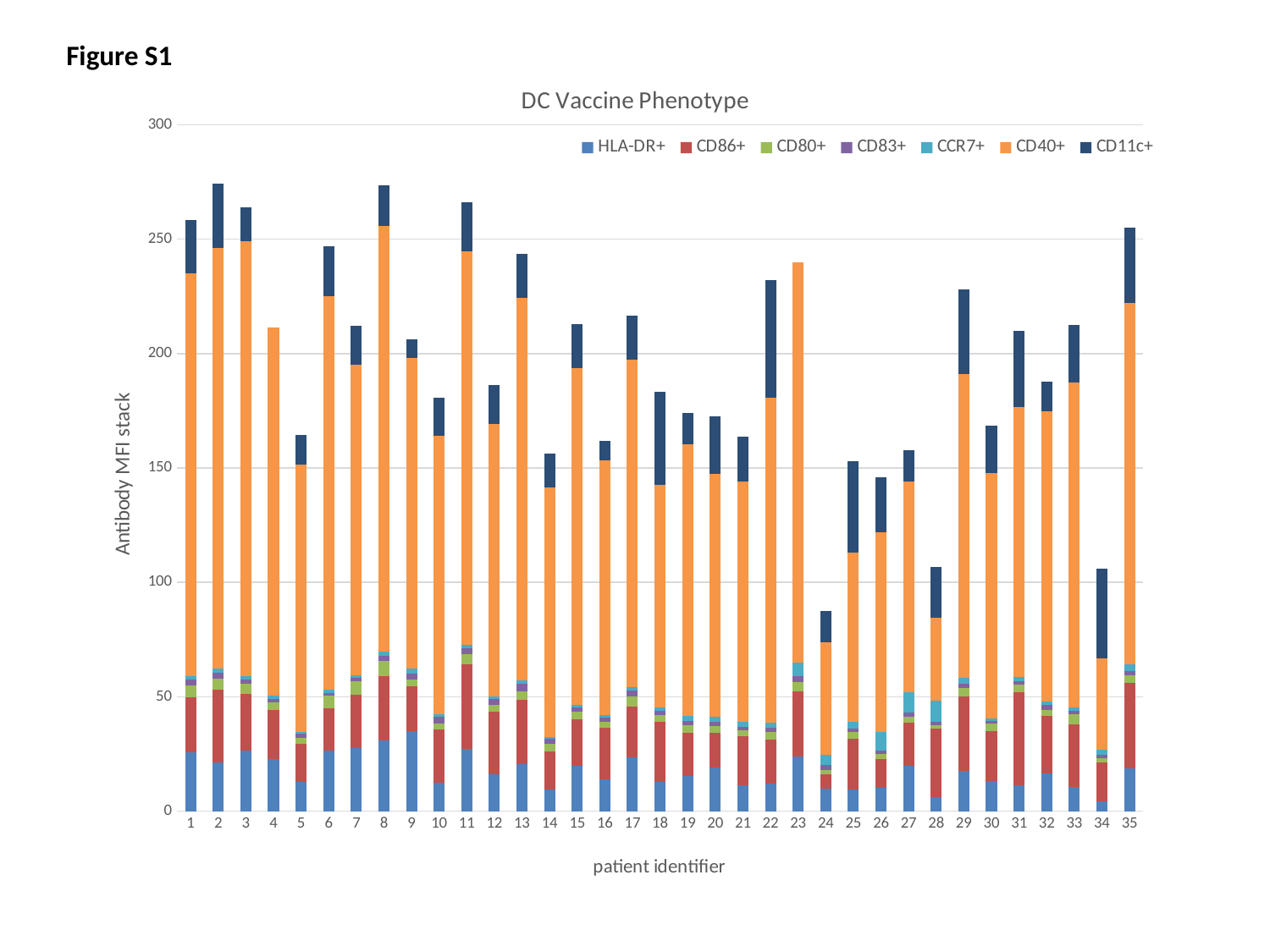

Figure S1
### Chart: DC Vaccine Phenotype
| Category | HLA-DR+ | CD86+ | CD80+ | CD83+ | CCR7+ | CD40+ | CD11c+ |
|---|---|---|---|---|---|---|---|
| 1 | 25.6 | 24.0 | 5.45 | 2.57 | 1.34 | 176.0 | 23.5 |
| 2 | 21.4 | 31.7 | 4.87 | 2.49 | 1.89 | 184.0 | 28.0 |
| 3 | 26.3 | 24.8 | 4.65 | 1.85 | 1.36 | 190.0 | 14.8 |
| 4 | 22.6 | 21.7 | 3.05 | 1.79 | 1.38 | 161.0 | None |
| 5 | 12.7 | 16.8 | 2.53 | 1.79 | 0.795 | 117.0 | 12.9 |
| 6 | 26.5 | 18.3 | 5.51 | 1.48 | 1.39 | 172.0 | 21.9 |
| 7 | 27.5 | 23.2 | 6.01 | 1.44 | 1.15 | 136.0 | 16.9 |
| 8 | 31.0 | 27.9 | 6.65 | 2.18 | 2.11 | 186.0 | 17.8 |
| 9 | 35.1 | 19.3 | 3.25 | 2.63 | 1.89 | 136.0 | 8.16 |
| 10 | 12.5 | 23.1 | 2.67 | 2.96 | 0.965 | 122.0 | 16.4 |
| 11 | 27.0 | 37.0 | 4.44 | 2.71 | 1.5 | 172.0 | 21.5 |
| 12 | 16.2 | 27.3 | 2.86 | 2.67 | 1.1 | 119.0 | 17.1 |
| 13 | 20.4 | 28.4 | 3.66 | 3.3 | 1.43 | 167.0 | 19.5 |
| 14 | 9.53 | 16.4 | 3.36 | 2.3 | 0.932 | 109.0 | 14.8 |
| 15 | 19.6 | 20.7 | 3.12 | 1.79 | 1.37 | 147.0 | 19.3 |
| 16 | 13.7 | 22.6 | 2.79 | 1.95 | 1.11 | 111.0 | 8.54 |
| 17 | 23.4 | 22.3 | 4.4 | 2.56 | 1.58 | 143.0 | 19.5 |
| 18 | 12.9 | 26.0 | 3.07 | 1.74 | 1.7 | 97.2 | 40.6 |
| 19 | 15.2 | 19.2 | 3.05 | 2.0 | 2.09 | 119.0 | 13.5 |
| 20 | 19.1 | 15.0 | 3.15 | 1.63 | 2.44 | 106.0 | 25.4 |
| 21 | 11.2 | 21.6 | 2.42 | 1.65 | 2.1 | 105.0 | 19.7 |
| 22 | 12.1 | 19.2 | 3.13 | 1.98 | 2.23 | 142.0 | 51.3 |
| 23 | 23.8 | 28.4 | 4.18 | 2.44 | 6.25 | 175.0 | None |
| 24 | 9.87 | 6.1 | 1.88 | 2.25 | 4.57 | 49.1 | 13.6 |
| 25 | 9.3 | 22.3 | 3.07 | 1.31 | 2.9 | 74.1 | 40.1 |
| 26 | 10.2 | 12.5 | 2.44 | 1.44 | 8.02 | 87.3 | 24.1 |
| 27 | 19.6 | 19.2 | 2.52 | 1.95 | 8.7 | 92.2 | 13.6 |
| 28 | 6.17 | 29.8 | 1.46 | 1.58 | 9.19 | 36.4 | 22.1 |
| 29 | 17.5 | 32.6 | 3.68 | 1.74 | 2.58 | 133.0 | 36.8 |
| 30 | 13.3 | 21.8 | 3.14 | 1.18 | 1.25 | 107.0 | 20.8 |
| 31 | 11.1 | 40.9 | 3.36 | 1.58 | 1.78 | 118.0 | 33.3 |
| 32 | 16.6 | 25.1 | 2.62 | 2.03 | 1.56 | 127.0 | 12.9 |
| 33 | 10.7 | 27.2 | 4.32 | 1.63 | 1.61 | 142.0 | 25.0 |
| 34 | 4.4 | 17.0 | 1.61 | 1.72 | 1.91 | 40.2 | 39.2 |
| 35 | 18.6 | 37.5 | 3.23 | 1.74 | 3.09 | 158.0 | 33.0 |

## Slide 2
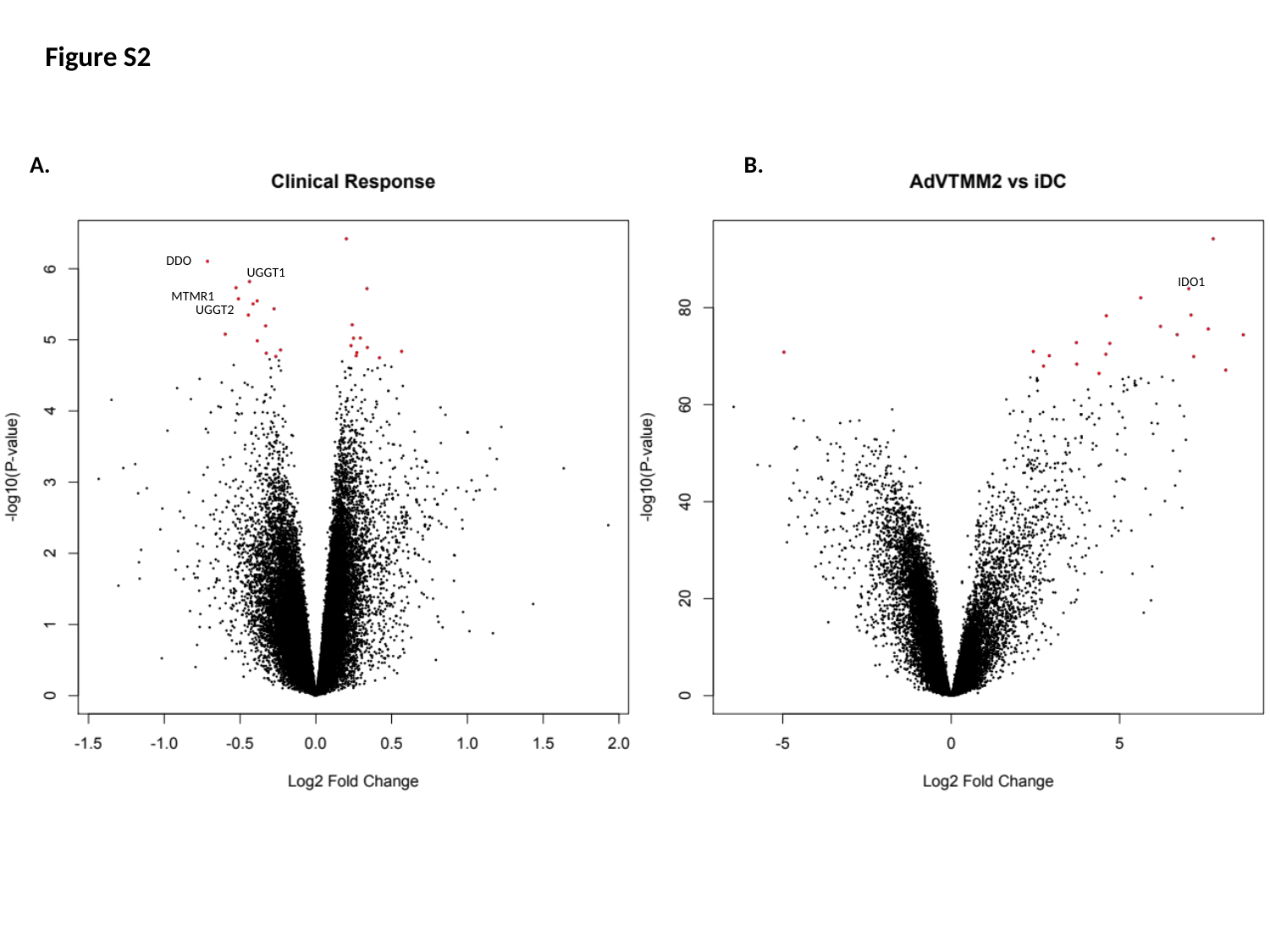

Figure S2
DDO
UGGT1
MTMR1
UGGT2
A.
IDO1
B.

## Slide 3
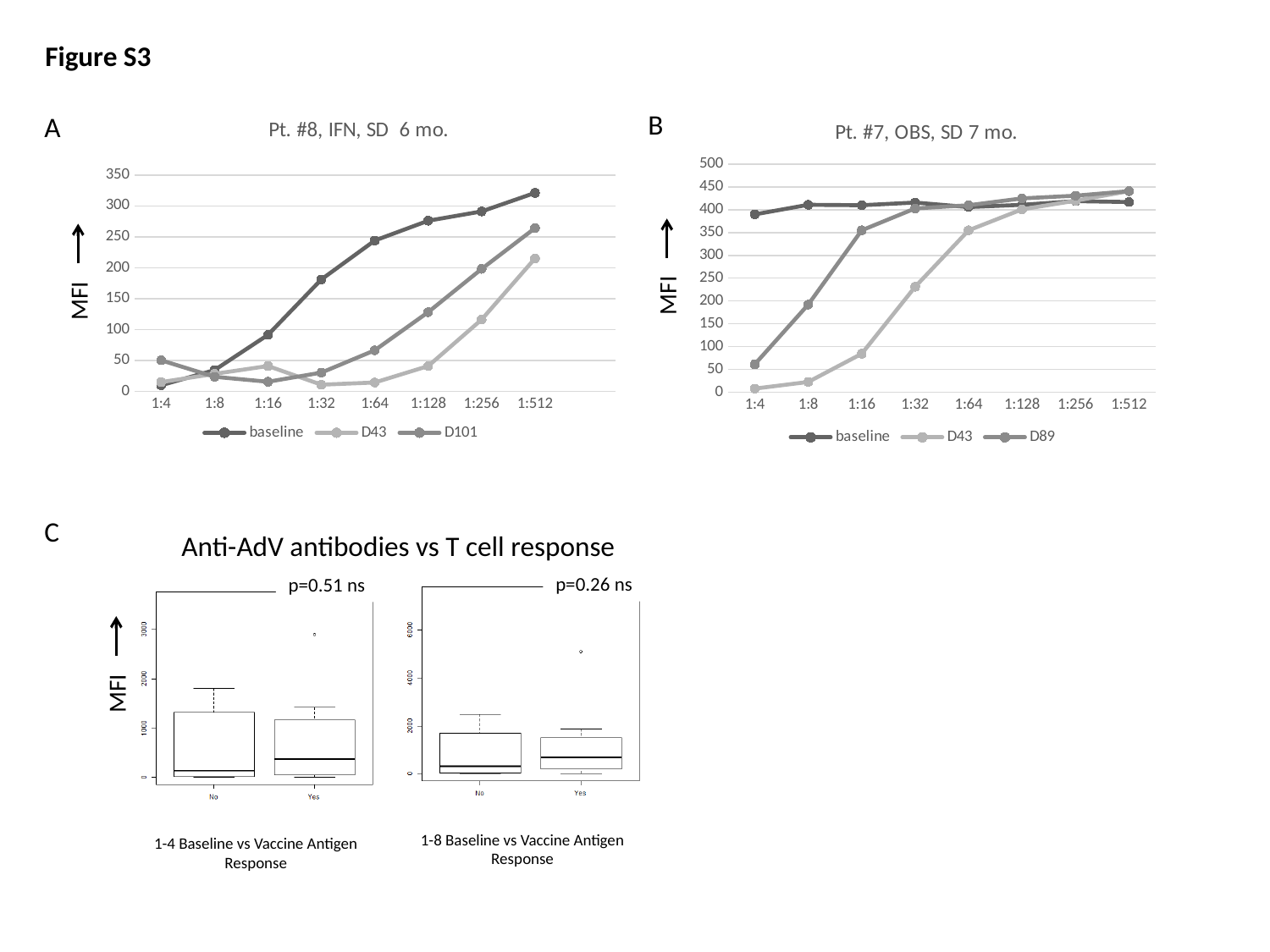

Figure S3
### Chart: Pt. #8, IFN, SD 6 mo.
| Category | baseline | D43 | D101 |
|---|---|---|---|
| 1:4 | 9.77 | 14.8 | 50.1 |
| 1:8 | 34.3 | 28.2 | 23.4 |
| 1:16 | 91.3 | 40.9 | 15.4 |
| 1:32 | 181.0 | 10.6 | 30.2 |
| 1:64 | 244.0 | 14.2 | 66.3 |
| 1:128 | 276.0 | 40.8 | 128.0 |
| 1:256 | 291.0 | 116.0 | 198.0 |
| 1:512 | 321.0 | 215.0 | 264.0 |B
A
### Chart: Pt. #7, OBS, SD 7 mo.
| Category | baseline | D43 | D89 |
|---|---|---|---|
| 1:4 | 390.0 | 7.59 | 61.0 |
| 1:8 | 411.0 | 22.2 | 192.0 |
| 1:16 | 410.0 | 84.0 | 355.0 |
| 1:32 | 416.0 | 231.0 | 403.0 |
| 1:64 | 406.0 | 355.0 | 410.0 |
| 1:128 | 411.0 | 401.0 | 425.0 |
| 1:256 | 419.0 | 420.0 | 431.0 |
| 1:512 | 417.0 | 441.0 | 441.0 |MFI
MFI
C
Anti-AdV antibodies vs T cell response
1-8 Baseline vs Vaccine Antigen Response
1-4 Baseline vs Vaccine Antigen Response
p=0.26 ns
p=0.51 ns
MFI
