# Supplementary material for: Multiple antigen-engineered DC vaccines with or without IFNα to promote antitumor immunity in melanoma
Source: J Immunother Cancer. 2019 Apr 24;7:113. doi: 10.1186/s40425-019-0552-x (PMC6480917; doi:10.1186/s40425-019-0552-x)
Supplement: Supplementary file 2 — Table S1. DC vaccine doses and cytokine production. Table S2. Inclusion and Exclusion Criteria. Table S3A. Worst Grade AE per Patient. Table S3B. Adverse Events of Grade 3 or 4. (DOCX 90 kb) [file 40425_2019_552_MOESM2_ESM.docx]

| **Supplementary Table 1: DC vaccine doses and cytokine production** | | | | | |  |
| --- | --- | --- | --- | --- | --- | --- |
| Patient Identifier | #DC  V1^1^ | V2^2^ | V3^3^ | Induced IL-12p70^4^ | Induced IL-10^5^ | Notes^6^ |
| 1 | 10e7 | 10e7 | 10e7 | 4,024 | 84 |  |
| 2 | 10e7 | 10e7 | 10e7 | 2,743 | 151 |  |
| 3 | 10e7 | 10e7 | 10e7 | 1,354 | 45 |  |
| 4 | 10e7 | 7.7x10e6 | (ND) | 1,205 | 219 |  |
| 5 | 10e7 | (ND) | (ND) | 4,108 | 691 |  |
| 6 | 10e7 | 6.9x10e6 | 7.5x10e6 | 195 | 844 |  |
| 7 | 10e7 | 9.3x10e6 | 9.9x10e6 | 730 | 112 |  |
| 8 | 10e7 | 8.2x10e6 | 7.2x10e6 | 7,177 | 3,267 |  |
| 9 | 10e7 | 5.1x10e6 | 5.0x10e6 | 55 | 435 |  |
| **10** | 10e7 | 7.8x10e6 | 5.1x10e6 | 348 | 73 |  |
| 11 | 7.5x10e6 | 6.6x10e6 | 5.5x10e6 | 384 | 279 |  |
| 12 | 5.1x10e6 | 5.6x10e6 | 6.1x10e6 | 231 | 439 |  |
| 13 | 10e7 | 5.1x10e6 | 6.8x10e6 | 3,009 | 283 |  |
| 14 | 7.5x10e6 | 6.6x10e6 | 5.3x10e6 | 833 | 203 |  |
| 15 | 10e7 | 5.9x10e6 | 6.2x10e6 | 391 | 3,169 |  |
| 16 | 10e7 | 5.6x10e6 | 7.3x10e6 | 250 | 100 |  |
| 17 | 10e7 | 3.6x10e6 | 4.3x10e6 | 147 | 350 |  |
| 18 | 4.3x10e6 | 5.7x10e6 | 5.5x10e6 | 70 | 188 |  |
| 19 | 10e7 | 7.1x10e6 | 9.4x10e6 | 58 | 317 |  |
| **20** | 10e7 | 6.1x10e6 | 6.1x10e6 | 124 | 197 |  |
| 21 | 10e7 | 5.4x10e6 | 7.4x10e6 | 347 | 369 |  |
| 22 | 10e7 | 8.4x10e6 | 5.2x10e6 | 205 | 511 | 59% granulocytes |
| 23 | 10e7 | 6.5x10e6 | 7.4x10e6 | 449 | 388 |  |
| 24 | 10e7 | 5.2x10e6 | 8.8x10e6 | 3 | 38 | 65% granulocytes |
| 25 | 10e7 | 8.2x10e6 | (ND) | 87 | 132 |  |
| 26 | 10e7 | 6.8x10e6 | 6.3x10e6 | 109 | 363 |  |
| 27 | 10e7 | 6.3x10e6 | 6.7x10e6 | 34 | 356 |  |
| 28 | 10e7 | 8.7x10e6 | 6.1x10e6 | 91 | 76 |  |
| 29 | 10e7 | 5.4x10e6 | 5.4x10e6 | 38 | 358 | 31% granulocytes |
| 30 | 10e7 | 5.2x10e6 | 5.9x10e6 | 886 | 58 |  |
| 31 | 10e7 | 6.8x10e6 | 5.4x10e6 | 150 | 219 |  |
| 32 | 10e7 | 6.5x10e6 | 8.5x10e6 | 129 | 265 |  |
| 33 | 10e7 | 7.5x10e6 | 7.4x10e6 | 324 | 113 |  |
| 34 | 10e7 | 5.4x10e6 | 6.2x10e6 | 168 | 107 |  |
| 35 | 10e7 | 6.3x10e6 | 5.4x10e6 | 171 | 339 |  |
| **Average** | 9.55x10e6 | 7.26x10e6 | | 875 | 432 |  |
| ^1^ cell count of fresh DC in first vaccine  ^2^ cell count of thawed vaccine 2  ^3^ cell count of thawed vaccine 3  ^4^ IL-12p70 in pg/mL secreted in 24 hrs. by 10e6 fresh DC vaccine #1 co-cultured with CD40L-expressing cells  ^5^IL-10 in pg/mL secreted in 24 hrs. by 10e6 fresh DC vaccine #1 co-cultured with CD40L-expressing cells  ND, not delivered due to PD.  ^6^Unusually high levels of granulocytes after monocyte elutriation are noted. | | | | | | |

| **Supplementary Table 2: Inclusion and Exclusion Criteria** |
| --- |
| ***Inclusion Criteria*** |
| Ability and willingness to give consent |
| Patients age 18 and older with recurrent, inoperable stage III, IV, M1a,b or c melanoma (any tumor thickness and any number of lymph node involvement, and in-transit metastases, or distant metastases) (AJCC) or resected stage IIIB-C or IV melanoma. Previously treated with any form of therapy provided the previous treatment was completed > 30 days prior to enrollment |
| Patients with measurable disease will ideally have at least 2 subcutaneous, intracutaneous, and accessible tumor deposits, lymph node or other site available for biopsy purposes. |
| Both men and women may be enrolled. Premenopausal females must have a negative pregnancy test. |
| ECOG Performance Status of 0 (n = 17) or ( n = 18) |
| No previous evidence of Class III or greater New York Heart Association cardiac insufficiency or coronary artery disease |
| No previous evidence of opportunistic infection |
| Adequate baseline hematological and organ function as assessed by the following laboratory values within 28 days prior to study entry:  Hemoglobin ≥9 g/dL  Granulocytes ≥2,000/mm3  Lymphocytes ≥500/mm3  Platelets >100,000/mm3  Serum Creatinine ≤1.5 X the ULN  AST, ALT, GGT, CPK, LDH, Alk phos ≤ 2.5 X the ULN  Serum Bilirubin ≤1.5 X ULN  In addition to study entry, the above hematological and organ function lab values along with the ECOG PS must be met prior to starting IFNα treatment. |
| Subjects must have normal coagulation parameters as measured by PT/PTT, unless the subject is on an anticoagulation therapy. |
| ***Exclusion Criteria*** |
| Subjects with acute infection: any acute viral, bacterial, or fungal infection which requires specific therapy. Acute therapy must have been completed more than 14 days prior to study treatment. |
| Hep B & C and HIV-infected patients, due to concerns in the ability to stimulate an effective immune response (determined by historical medical data). |
| Subjects with acute medical problems such as ischemic heart or lung disease that may be considered an unacceptable anesthetic or operative risk. |
| Subjects with any underlying conditions which would contraindicate therapy with study treatment (or allergies to reagents). |
| Subjects with organ allografts. |
| Subjects must be free of known brain metastases by contrast-enhanced CT/MRI scans or have successfully-treated brain metastases and be asymptomatic for more than 1 month. |
| Patients requiring immunosuppressive therapy for comorbid conditions. |
| Concomitant Medication and Treatment: All allowed medications or treatments should be kept to a minimum and recorded. All questions regarding concomitant medications should be referred to the Investigator. |
| Long-term concurrent medications and/or treatments **not allowed:** Corticosteroids, chemotherapy, cyclosporine A. Short term (approximately 1 week) use of topical, low-dose or inhaled steroids may be allowed at the discretion of the investigator. Injectables **not** allowed. |

| **Supplemental Table 3A: Worst Grade AE per Patient** | | |
| --- | --- | --- |
| Grade | Frequency | Percent |
| 1 | 9 | 50 |
| 2 | 2 | 11.11 |
| 3 | 6 | 33.33 |
| 4 | 1 | 5.56 |
| Total | 18 | 100 |

**Supplemental Table 3**

| **Supplemental Table 3B: Adverse Events of Grade 3 or 4** | | | |
| --- | --- | --- | --- |
| Toxicity | Grade 3 | Grade 4 | Agent attribution |
| Fatigue | 5 | 0 | Interferon Alpha2b (Intron A) |
| GGT increased | 1 | 0 | Interferon Alpha2b (Intron A) |
| Nausea | 1 | 0 | Interferon Alpha2b (Intron A) |
| Neutrophil count decreased | 3 | 1 | Interferon Alpha2b (Intron A) |
| White blood cell decreased | 2 | 0 | Interferon Alpha2b (Intron A) |
